# Delineating the impacts of air temperature and humidity for endurance exercise

**DOI:** 10.1113/EP090969

**Published:** 2022-12-20

**Authors:** Elliott J. Jenkins, Holly A. Campbell, Jason K. W. Lee, Toby Mündel, James D. Cotter

**Affiliations:** ^1^ School of Physical Education Sport and Exercise Sciences University of Otago Dunedin New Zealand; ^2^ Cardiff School of Sport and Health Sciences Cardiff Metropolitan University Cardiff UK; ^3^ Department of Surgical Sciences Dunedin School of Medicine University of Otago Dunedin New Zealand; ^4^ Department of Physiology National University of Singapore Singapore; ^5^ Heat Resilience and Performance Centre Yong Loo Lin School of Medicine National University of Singapore Singapore; ^6^ Human Potential Translational Research Programme Yong Loo Lin School of Medicine National University of Singapore Singapore; ^7^ Department of Physiology Yong Loo Lin School of Medicine National University of Singapore Singapore; ^8^ Campus for Research Excellence and Technological Enterprise (CREATE) Singapore; ^9^ School of Sport Exercise and Nutrition Massey University Palmerston North New Zealand

**Keywords:** absolute humidity, air temperature, exercise physiology, heat stress

## Abstract

Many studies have reported that ambient heat stress increases physiological and perceptual strain and impairs endurance exercise, but effects of air temperature per se remain almost unexamined. Most studies have used matched *relative* humidity, thereby exponentially increasing *absolute* humidity (water content in air) concurrently with temperature. Absolute (not relative) humidity governs evaporative rate and is more important at higher work rates and air temperatures. Therefore, we examined the independent effects of air temperature and humidity on performance, thermal, cardiovascular and perceptual measures during endurance exercise. Utilizing a crossover design, 14 trained participants (7 females) completed 45 min fixed‐intensity cycling (70% ${\dot{V}}_{O_{2}\mathrm{peak}}$) followed by a 20‐km time trial in each of four environments: three air temperatures at matched absolute humidity (Cool, 18°C; Moderate, 27°C; and Hot, 36°C; at 1.96 kPa, air velocity ∼4.5 m/s), and one at elevated humidity (Hot Humid, 36°C at 3.92 kPa). Warmer air caused warmer skin (0.5°C/°C; *P* < 0.001), higher heart rate (1 bpm/°C; *P* < 0.001), sweat rate (0.04 l/h/°C; *P* < 0.001) and thermal perceptions during fixed‐intensity exercise, but minimally affected core temperature (<0.01°C/°C; *P* = 0.053). Time‐trial performance was comparable between Cool and Moderate (95% CI: –1.4, 5.9%; *P* = 0.263), but 3.6–6% slower in Hot (95% CI: ±2.4%; *P* ≤ 0.006). Elevated humidity increased core temperature (*P* < 0.001), perceived temperature and discomfort but not skin temperature or heart rate, and reduced mean blood pressure (*P* = 0.046) during fixed‐intensity exercise. Elevated humidity impaired time‐trial performance by 3.4% (95% CI: ±2.2%; *P* = 0.006). In conclusion, these findings quantify the importance of absolute humidity alongside air temperature when exercising under severe heat stress.

## Abstract (highlights)

- **What is the central question of this study?**
  What are the independent effects of air temperature and humidity on performance, physiological and perceptual responses during endurance exercise?
- **What is the main finding and its importance?**
  When examined independently, elevated air temperature increased heat strain and impaired aerobic exercise performance, but to a lesser extent than has been reported previously. These findings highlight the importance of absolute humidity relative to temperature when exercising or working under severe heat stress.

## 1 INTRODUCTION

Humans excel at sustained physical exertion in dry heat; we are relatively hairless, almost entirely covered in high‐output eccrine sweat glands, and typically exercise moving upright through the air. Evaporating sweat is our most powerful mode of heat loss, and in hot conditions becomes our only real means for it, thereby enabling sustained exercise or work. The rate of evaporation from exposed and fully wetted skin depends almost entirely on the air's absolute humidity (i.e., water content (g/m^3^) or vapour pressure (kPa)), which is increasingly important to heat balance and thus performance and tolerance at higher air temperatures or work rates.

Many studies have shown, based on controlled lab experiments or field observations (e.g., of major marathons), that sustained/aerobic exercise performance is impaired in hot environments (Galloway & Maughan, 1997; Montain et al., 2007). Unfortunately, almost all lab studies have used matched *relative* humidity (RH; e.g., 50% of the maximal water content possible at that temperature) and thereby also used an exponentially higher absolute humidity in their ‘warmer environments’. Thus, it remains unresolved what effect air temperature per se has on sustained exercise performance or its underlying physiology and psychophysiology, particularly for conditions in which evaporation is more important, that is, higher air temperature or work rate.

Establishing the role of air temperature per se is important in many theoretical and practical contexts (Brotherhood, 2008; Lei et al., 2020). Theoretical contexts include prediction of safety or tolerance times, while practically, if air temperature climbed from 10°C to 35°C during a day, absolute humidity may be relatively stable at ∼1 kPa, causing RH to drop substantially, from 82% to 18%. To match the RH would require 4.6 times more water being added to the warmer air, with a corresponding reduction in the vapour pressure gradient for evaporation from saturated skin to that air.

To our knowledge, only one recent study, Lei et al. (2020), has utilised matched absolute humidity (2.8 ± 0.1 kPa) across multiple air temperatures (29.2 ± 0.2°C and 34.9 ± 0.2°C), finding no significant performance decrements of a warmer environment in trained male cyclists, despite higher skin temperatures. However, as the temperature difference between conditions was narrow, and exposure time was <40 min, the likelihood of revealing the effect of air temperature per se on performance, thermal and cardiovascular responses was limited. Bright et al. (2021), also acknowledged the confounding effects of humidity on exercise performance in the heat, but addressed a different question, examining the effects of dry‐bulb temperature on self‐paced exercise performance while attempting to minimise the saturated skin‐to‐air vapour pressure (*P*_sk,sat_ – *P*_a_) *gradient* (i.e., evaporative potential) between four thermal conditions. They found that at lower air temperatures performance was unaffected when evaporative capacity was maintained, but at higher temperatures, the uncompensability of the environment alongside reductions in dry heat loss increased thermal, cardiovascular and perceptual strain, thereby impairing performance. Conversely, the importance of humidity has been examined at a fixed air temperature and was shown to increase heat storage, thermal discomfort and physiological strain, reducing endurance capacity in a warm low‐airflow environment (Maughan et al., 2012; Muhamed et al., 2016).

The aim of this study, therefore, was to determine the independent effects of air temperature and humidity on performance, thermal, cardiovascular, and perceptual effects of endurance exercise. The emphasis here was to use a wide and relevant range of air temperatures matched for absolute humidity, and examine humidity effects only at high air temperature, hence the design described in Section 2.2. We anticipated that elevated air temperature would increase heat strain and impair performance but to a lesser extent than reported previously. This working hypothesis was tested by quantifying the effects of air temperature with and without matched absolute humidity, as described in Sections 2.8 and 3.6.

## 2 METHODS

### 2.1 Ethical approval

Procedures were approved by the University of Otago Human Ethics Committee (Health) (Project No. H21/055) and conformed to the *Declaration of Helsinki* (2013), other than prior registration in a database. Written, informed consent was obtained from each participant prior to testing.

### 2.2 Experimental design

**FIGURE 1:**
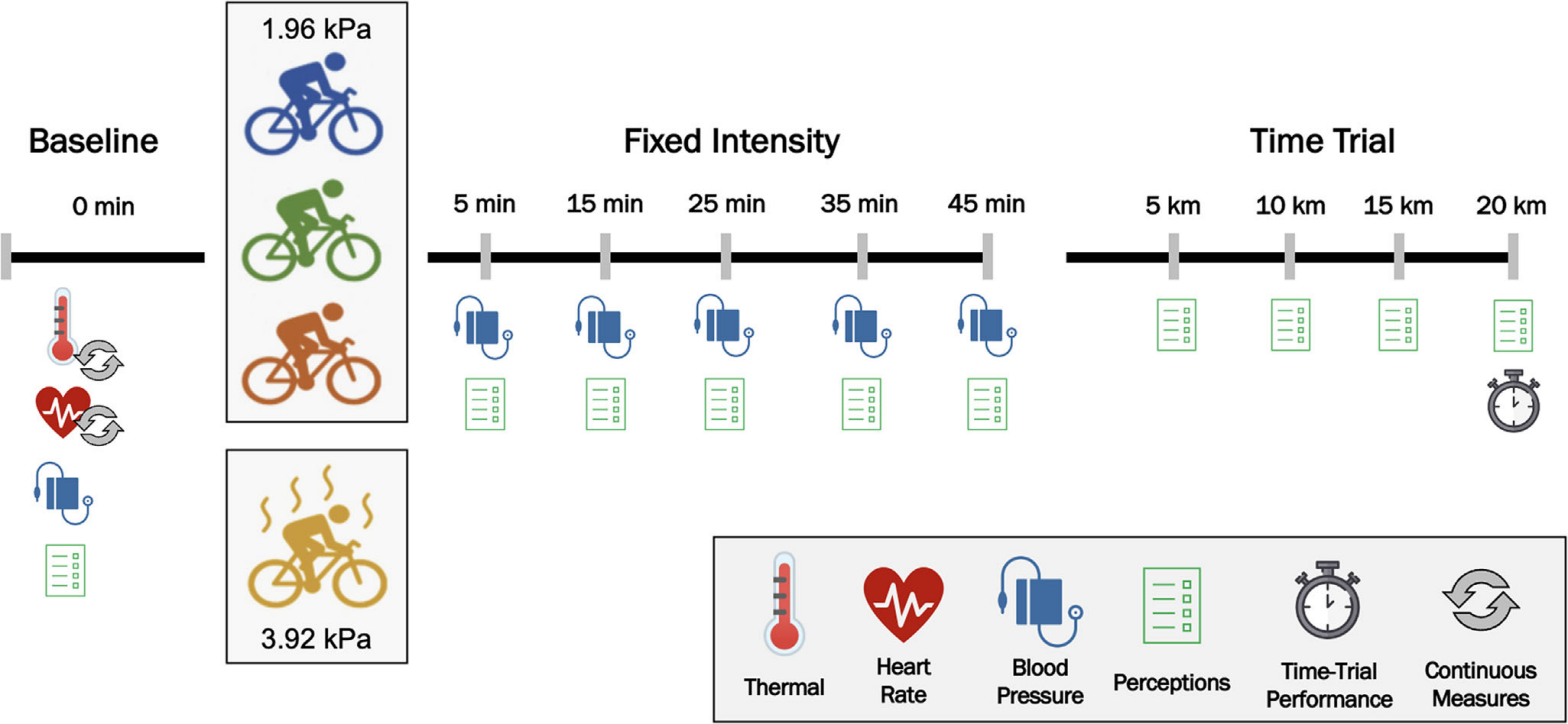
Schematic representation of experimental procedures across four environmental conditions: Cool, Moderate, Hot (18, 27, and 36°C all at 1.96 kPa) and Hot Humid (36°C at 3.92 kPa). Measures: thermal: rectal temperature, skin temperature and sweat rate kinetics; perceptions: rate of perceived exertion, thermal sensation, thermal discomfort and feeling scale (affect); time‐trial performance: time taken to cycle 20 km

A within‐participants, randomised‐crossover design was used to compare performance, thermal, cardiovascular and perceptual measures across four environmental conditions: three at different air temperatures but matched absolute humidity (i.e., 18, 27 and 36°C, at 1.96 kPa, or 95%, 55% and 33% RH, respectively), and one at an elevated humidity (36°C, at 3.92 kPa, or 66% RH). The design is shown in Figure 1. Each trial consisted of 45 min of fixed‐intensity cycling at 70% ${\dot{V}}_{O_{2}\mathrm{peak}}$, followed by a 20‐km time trial (TT). Air velocity was maintained at 4.5 m/s, provided by a large‐diameter (65 cm) fan situated 0.9 m in front of participants’ handlebars. Primary measurements included thermal, cardiovascular and perceptual measures, collected during both phases, and performance time. Trials were separated by 1 week to minimise potential for fatigue or acclimation, and conducted at the same time of day (±0.5 h) within participants to minimise circadian rhythm effects. All testing was completed between July and October within the environmental control laboratory at the School of Physical Education, Sport and Exercise Sciences (University of Otago, Dunedin, New Zealand).

### 2.3 Participants

Fourteen participants (7 males and 7 females, aged 28 ± 13 years, height 170 ± 7 cm, body mass 67 ± 7 kg, body surface area 1.78 ± 0.12 m^2^) completed the study. Participants were aerobically fit (cycling ${\dot{V}}_{O_{2}\mathrm{peak}}$: 55 ± 9 ml/min/kg), exercising regularly (≥3 times/week), familiar with maximal exercise exertion and unacclimated to heat. Prior to testing sessions participants were asked to: (i) refrain from strenuous exercise and alcohol for 24 h; (ii) standardise their previous two meals and caffeine consumption; and (iii) be well hydrated. Females were tested only when in the luteal phase of their naturally occurring menstrual cycle, or at regular weekly intervals during the active pill phase if they were taking an oral contraceptive pill.

### 2.4 Preliminary measures and familiarisation

Following basic anthropometric measures (height and body mass), participants underwent a standard protocol for the measurement of ${\dot{V}}_{O_{2}\mathrm{peak}}$ in a temperate environment (21°C, 50% RH). An incrementing‐to‐maximum exercise test was performed on an electromagnetically braked cycle ergometer (Velotron, RacerMate inc, Seattle, WA, USA), with online respiratory gas analysis (CPET, Cosmed, Rome, Italy). Gas analysers and airflow turbines were calibrated prior to each test. Power output was set initially at 100 W for 3 min, then increased by 20 W/min for females, or 30 W/min for males at 1‐min intervals thereafter until voluntary exhaustion. ${\dot{V}}_{O_{2}\mathrm{peak}}$ was taken as the maximum value recorded over a 15‐s average. Following appropriate recovery time, participants were invited to the environmental chamber for a familiarisation session consisting of a shortened exercise protocol (20‐min fixed‐intensity cycle, 5‐km TT in 22°C, 50% RH conditions) with full measurement procedures to minimise learning effects. Seat height and saddle position were measured for each participant and standardised thereafter.

### 2.5 Experimental procedure

All four experimental trials utilised the same protocol. Following a verbal check of compliance with standardisation requirements, participants emptied their bladder for the measurement of urine specific gravity (USG). If USG was >1.020, participants drank 500 ml of water. Participants then inserted a rectal thermistor, had body mass measured, and sat in a temperate environment (19.5°C, 50% RH) to be instrumented for measurement of heart rate, skin temperature (*T*_sk_) and local sweat rate. Following a minimum of 5 min of quiet rest, baseline measurements were taken for core temperature (*T*_c_), *T*_sk_, heart rate, blood pressure and thermal perceptions.

Participants then entered the environmental chamber and sat on the cycle ergometer, at which point the sweat capsule was ventilated with dry air, the fan was turned on, and they began cycling. Exercise was at a fixed relative intensity of 70% ${\dot{V}}_{O_{2}\mathrm{peak}}$ for 45 min, throughout which, power output, *T*_c_, *T*_sk_, heart rate and local sweat rate were measured continuously, while blood pressure and perceptual measures were collected at 5 min and then at 10‐min intervals. Following this bout, participants rested for ∼90 s while the ergometer was switched to TT mode, after which they completed 20 km as fast as possible. Aforementioned continuous measures remained so, while perceptual measures were collected at 5‐km intervals (Figure 1). Feedback during the TT was only distance travelled and current gear. Participants received no verbal encouragement. Music was self‐selected and kept similar across experimental conditions. Cool tap water (∼19°C) was provided for drinking ad libitum throughout the exercise protocol, with total fluid consumption monitored throughout. Participants were asked to attempt to limit dehydration to less than 2% to avoid possible confounding effects.

Upon completing the TT, participants exited the environmental chamber and were deinstrumented before they dried themselves off and had their post‐exercise semi‐nude body mass measured. They then emptied their bladder for determination of USG and urine volume.

### 2.6 Measurements

Height and mass were measured using a standard stadiometer and electronic scales (D1–10, Wedderburn, Dunedin, New Zealand; calibrated to 0.02 kg), respectively. USG was measured with a handheld refractometer (Uricon‐N, Atago, Tokyo, Japan).

Rectal temperature was measured using a flexible thermistor (Mon‐a‐therm, General Purpose Thermistors, 400 series, Mallinckrodt Inc., St Louis, MO, USA), self‐inserted to a standardised depth (∼15 cm). Skin temperature was measured using insulated thermistors (2.3K3A1B Thermistor NTC, Betatherm, Galway, Ireland) taped (Sport premium rigid, USL, Auckland, New Zealand) to six right‐side sites: subscapular, upper chest, dorsal forearm, forehead, anterior thigh and lateral calf. Temperatures were averaged according to surface area weightings to provide a mean *T*_sk_. All temperatures were recorded at 15‐s intervals using a portable logger (Squirrel SQ2010, Grant Instruments, Cambridge, UK).

Heart rate was measured continuously (beat‐to‐beat) from the R–R period of ventricular depolarisation (T31, Polar, Kempele, Finland), and converted to digital signal at 1 kHz (PowerLab, ADInstruments, Otago, New Zealand) for storage on computer software (LabChart 4.20, ADInstruments). Brachial arterial blood pressure was measured in duplicate using a manual sphygmomanometer and stethoscope. Blood pressure measurements were obtained by the same researcher for each participant across all of their conditions.

Sweat rate kinetics were measured using a ventilated capsule system, from one capsule (19 mm ID) taped to the left subscapular in a standardised location for each participant. Capsules were ventilated at a continuously measured flowrate (flow sensor; Honeywell AWM5101, Freeport, IL, USA) of ∼0.20 L/min from bottled dry air. Post‐capsular airstream was sampled for humidity (resistance hygrometer; Honeywell HIH 3605) and temperature (National Semiconductors LM35CAH, Hong Kong). Digital outputs from this system were obtained (PowerLab hardware 8e, ADInstruments) and recorded at 1 Hz on LabChart software. Sweat onset was determined from visual inspection of the sweat trace. Whole‐body sweat rate was estimated from change in body mass and adjusted for fluid consumed and urine excreted, both measured via electronic platform balance (TX4202L, Shimadzu, Kyoto, Japan).

Perceptual measures collected were: rating of perceived exertion (RPE) (range, 6–20, no exertion – maximal exertion; Borg, 1970); thermal sensation (range, 1–13, unbearably cold – unbearably hot); thermal discomfort (range, 1–10, comfortable – extremely uncomfortable; both adapted from Gagge et al., 1967) and affect (range, +5 to –5, very good – very bad; Hardy and Rejeski, 1989).

### 2.7 Data analysis

Body surface area was estimated using the formula of Du Bois and Du Bois (1916).

Mean *T*_sk_ was estimated from surface area weightings, adapted from Hardy et al. (1938): (0.175 × Subscapular) + (0.175 × Chest) + (0.19 × Forearm) + (0.07 × Forehead) + (0.19 × Thigh) + (0.2 × Calf).

Participants’ heart rate was expressed as a percentage of their maximum (i.e., %HR_max_) – as recorded from their incrementing exercise test. Mean arterial blood pressure (MAP) was estimated as the sum of two‐thirds diastolic blood pressure (DBP) and one‐third systolic blood pressure (SBP).

### 2.8 Statistical analysis

All data were collated using Microsoft Excel. Descriptive statistics are reported as means ± standard deviation (SD). Data were analysed inferentially using repeated measures, mixed models analysis of variance and Student's paired *t*‐test to explore differences between air temperatures and humidities where relevant, with α set at 0.05 for each analysis. Main time effects were not pursued *post hoc*, as the effect of exercise over time has already been well established. While presented figures and tables depict all four environmental conditions together, statistical analyses and comparisons were made only for those three conditions independently altering temperature (matching for absolute humidity), and for those two conditions independently manipulating humidity (controlling for temperature). Furthermore, fixed intensity and self‐paced periods were analysed separately.

For Section 3.6, statistical analyses were run on all four environmental conditions together to convey the differences between the present study and previous studies that have investigated the effect of temperature with the confounding effects of RH, thus directly addressing our research hypothesis. Differences are reported as means and 95% confidence intervals (lower limit, upper limit) for those comparisons of interest. Statistical analyses and graphing were completed using Prism Version 9.20 (GraphPad Software, San Diego, CA, USA).

## 3 RESULTS

Results presented are for 14 participants unless stated otherwise. Environmental parameters were stable at target levels in all trials except for the Cool condition, in which absolute humidity was 11% (0.21 kPa) below its set point. The experimental trials are referred to as Cool (denoting 18.1 ± 0.0°C, 1.75 ± 0.05 kPa or 84 ± 3% RH), Moderate (denoting 27.1 ± 0.0°C, 1.99 ± 0.01 kPa or 56 ± 0% RH), Hot (denoting 36.1 ± 0.1°C, 2.00 ± 0.01 kPa or 33 ± 0% RH) and Hot Humid (denoting 36.2 ± 0.0°C, 3.99 ± 0.01 kPa or 67 ± 0% RH).

### 3.1 Participant compliance

All participants reported compliance with the outlined standardisations, and completed the study, finishing the TT in each of the four environmental conditions. One participant was unable to complete the 45‐min fixed‐intensity period in Hot and Hot Humid, stopping cycling due to exhaustion at 38 and 26 min, respectively. In this event, the participant was provided with ∼90 s rest prior to beginning the TT. This participant's data are excluded from the fixed‐intensity results but included in the TT. Significant performance findings were not any different regardless of this participant's inclusion or exclusion from TT data.

### 3.2 Performance

**FIGURE 2:**
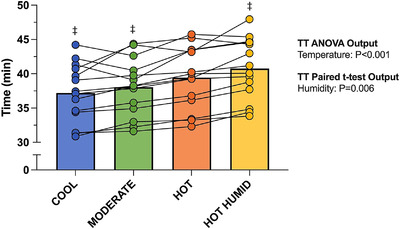
Mean and individual time‐trial performance in each thermal environment. TT, time trial. ^‡^Significant difference from Hot (*P* < 0.05). Comparisons were made only between the three air temperatures (three bars on left), and between the two humidities (two bars on right). These comparisons and style of notations are used on all tables and figures. *n* = 14

Ambient temperature significantly affected TT performance (*P* < 0.001; Figure 2). Performance time was comparable between Cool and Moderate (95% CI: −0.5, 2.2; *P* = 0.263) but was faster in Cool than in Hot, by 2.2 min (95% CI: 1.4, 3.1; *P* < 0.001), and in Moderate than in Hot, by 1.4 min (95% CI: 0.4, 2.3; *P* = 0.006). In relative terms, these performance impairments were 6% and 3.6%, respectively.

Elevated humidity at 36°C resulted in a slower TT performance, by 1.3 min (95% CI: 0.5, 2.2; *P* = 0.006; Figure 2), or 3.4%. The effects of both temperature (*P* < 0.001) and humidity (*P* = 0.004) on mean power output throughout the TT's duration were consistent with these findings (Table 1).

**TABLE 1:** Mean power (W) produced during the initial, middle and final sections and throughout the time trial in each of four environments

Stage	Cool	Moderate	Hot	Hot Humid
0−2 km	195 (54)^‡^	183 (51)^‡^	168 (52)	151 (42)^‡^
6−15 km	191 (60)^‡^	181 (51)^‡^	166 (52)	151 (46)^‡^
18−20 km	226 (72)^‡^	211 (65)^‡^	192 (59)	183 (55)^‡^
Throughout	198 (61)^‡^	185 (53)^‡^	170 (53)	156 (45)^‡^

Data are means (SD). Sections were selected to represent the pace sustained initially (0–2 km), during the middle (6–15 km), in the final end spurt (18–20 km), and throughout (0–20 km) the time‐trial. ^‡^Significant difference from Hot (*P* < 0.05). *n* = 14.

### 3.3 Thermal

#### 3.3.1 Core temperature

**FIGURE 3:**
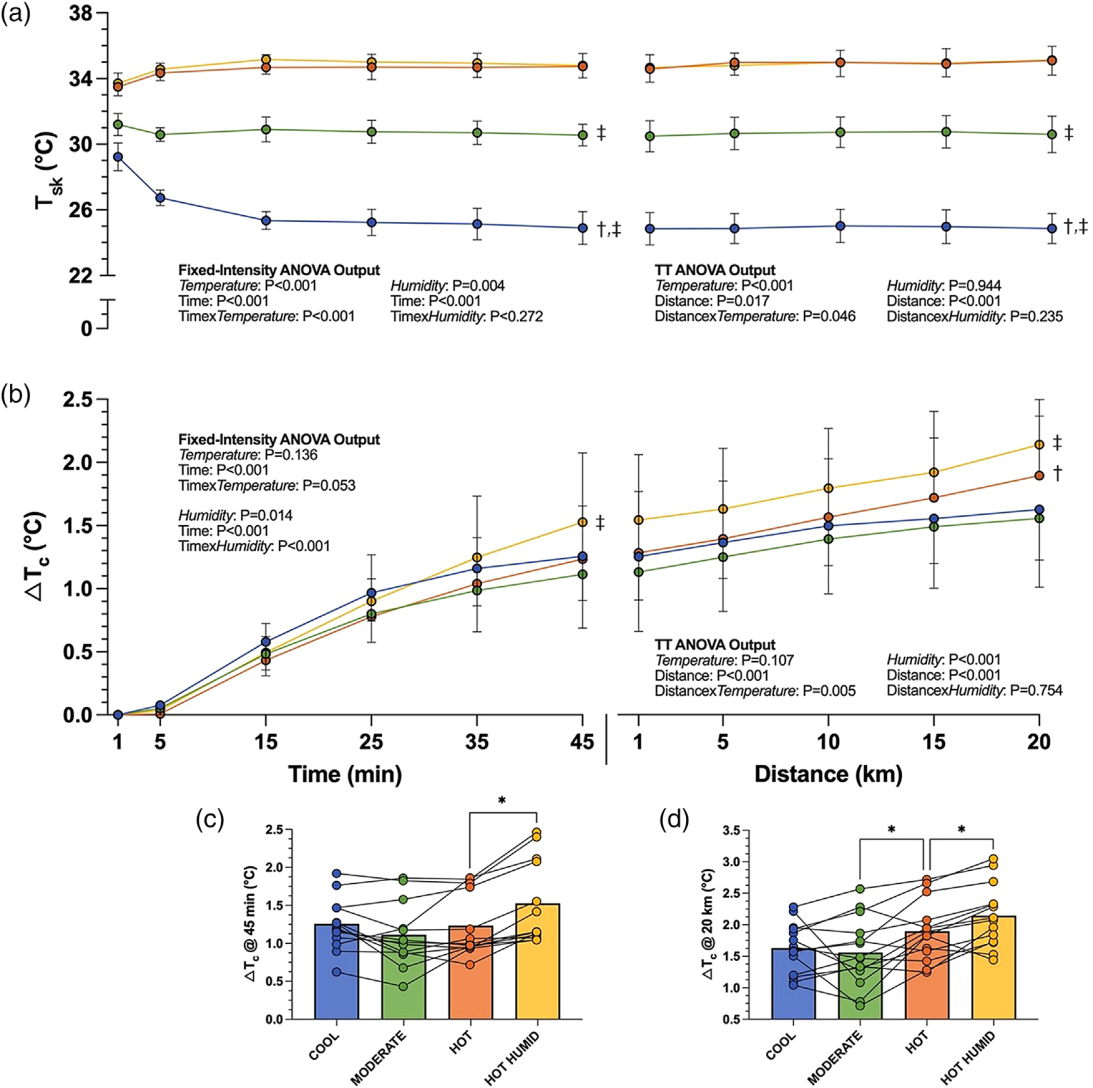
(a, b) Change in mean skin temperature (a) and change in mean core temperature (b) (±SD) throughout 45 min of fixed‐intensity cycling followed by a 20‐km time trial. (c, d) Individual changes in core temperature data shown for 45 min (c) and 20 km (d) time points. ^†,‡^Significant difference from Moderate (†) or Hot (‡) at end of 45‐min fixed‐intensity period or end of TT (*P* < 0.05). *Significant difference between conditions (*P* < 0.05). *n* = 13 for fixed intensity, *n* = 14 for TT. Δ*T*_c_, change in mean core temperature (rectal); *T*_sk_, mean skin temperature; TT, time trial

From baseline to end of exercise, *T*_c_ rose by 1.6 ± 0.4°C in Cool, 1.6 ± 0.6°C in Moderate, and 1.9 ± 0.5°C in Hot. Environmental temperature did not reliably affect the Δ*T*_c_ during the fixed‐intensity cycle (interaction: *P* = 0.053; air temperature: *P* = 0.136) but did by the end of the TT (interaction: *P* = 0.016). Specifically, no differences in Δ*T*_c_ from baseline to TT completion were evident between Cool and Moderate (*P* = 0.834) or between Cool and Hot (*P* = 0.078), whereas Δ*T*_c_ in Hot was 0.3°C larger than in Moderate (95% CI: 0.1, 0.6; *P* = 0.020; Figure 3).

The Hot Humid environment yielded the highest Δ*T*_c_ within the fixed‐intensity exercise (1.5 ± 0.5°C), and upon completion of the TT (2.1 ± 0.5°C). Unlike the effect of air temperature, humidity significantly affected Δ*T*_c_ from baseline across both the fixed‐intensity period (interaction: *P* < 0.001) and by the end of the TT (*P* = 0.004). *Post hoc* analyses revealed that Hot Humid resulted in a significantly larger Δ*T*_c_ compared to Hot from 25 min (*P* = 0.007) onward, being 0.3°C higher by 45 min (95% CI: 0.2, 0.4; *P* < 0.001), and remaining 0.2°C higher at 20 km (95% CI: 0.1, 0.4; *P* = 0.004) despite the lower work rate. The mean *T*_c_ at completion of the TT in Hot Humid was 39.1 ± 0.5°C (Figure 3).

#### 3.3.2 Skin temperature

Mean *T*_sk_ during exercise was 25.4 ± 0.7°C in Cool, 30.8 ± 0.8°C in Moderate and 34.7 ± 0.6°C in Hot. Air temperature had a significant effect on *T*_sk_ throughout the fixed‐intensity cycle (interaction: *P* < 0.001) and by the end of the TT (*P* < 0.001).

Humidity did not affect the *T*_sk_ or its time course during the fixed‐intensity exercise period (*P* = 0.272), though Hot Humid averaged 0.3°C warmer than Hot during the TT (95% CI: 0.1, 0.4; *P* = 0.004, Figure 3).

#### 3.3.3 Sweat rate and sweating kinetics

Environmental temperature affected whole‐body sweat rate, sweat onset time, and the *T*_c_ at which that onset occurred (all *P* < 0.001). In comparison to Cool, mean sweat rate was 0.29 L/h higher in Moderate (95% CI: 0.15, 0.43; *P* < 0.001) and 0.66 L/h higher in Hot (95% CI: 0.46, 0.85; *P* < 0.001). Sweating onset occurred 5.3 min earlier in Hot than in Cool (95% CI: 3.1, 7.4; *P* < 0.001), and 3 min earlier than in Moderate (95% CI: 1.4, 4.5; *P* < 0.001). Despite this, the *T*_c_ at sweat onset was not lower in Hot than in Moderate (*P* = 0.080), but was lower than in Cool, by 0.3°C (95% CI: 0.2, 0.5; *P* < 0.001).

Humidity did not affect sweat rate (*P* = 0.512) or the *T*_c_ at sweat onset (*P* = 0.797), despite onset occurring earlier in Hot Humid, by 1.2 min (95% CI: 0.4, 2.0; *P* = 0.007; Table 2).

**TABLE 2:** Sweat rate, sweating kinetics and hydration during exercise in four different environments

	Cool	Moderate	Hot	Hot Humid
Sweat rate (L/h)	0.41 (0.17)^†,‡^	0.70 (0.28)^‡^	1.07 (0.35)	1.03 (0.36)
Sweat onset (min)	9.6 (3.0)^†,‡^	7.3 (1.6)^‡^	4.3 (1.6)	3.1 (1.7)^‡^
*T*_c_ threshold (°C)	37.3 (0.4)^‡^	37.1 (0.4)	36.9 (0.4)	37.0 (0.4)
Fluid intake (ml)	470 (310)^†,‡^	670 (329)^‡^	1022 (393)	1143 (474)
Dehydration (%)	0.5 (0.4)^‡^	0.8 (0.5)	1.0 (0.5)	0.7 (0.6)

Data are means (SD). Sweat rate calculated over duration of protocol's entirety (45‐min fixed‐intensity cycling followed by a 20‐km time‐trial). *T*_c_ threshold indicates the core temperature at which the onset of sweating occurred. Dehydration expressed as a percentage of initial body weight. ^†^Significant difference from Moderate (*P* < 0.05); ^‡^significant difference from Hot (*P* < 0.05). *n* = 14. *T*_c_, core temperature (rectal).

Fluid consumption during exercise was affected by air temperature (*P* < 0.001), but not by humidity (*P* = 0.255; Table 2). Fluid intake was lower in Cool than in Moderate (by 200 ml 95% CI: 45, 355; *P* = 0.012) and Hot (by 553 ml 95% CI: 325, 780; *P* < 0.001), and lower again in Moderate than in Hot (by 353 ml 95% CI: 116, 590; *P* = 0.005). Dehydration was limited to within 2% of body mass across 55/56 trials, though appeared to be affected by air temperature (*P* = 0.006), being mitigated better in Cool than in Hot (by 0.5% 95% CI: 0.1, 0.8; *P* = 0.005; Table 2).

### 3.4 Cardiovascular

#### 3.4.1 Heart rate

**FIGURE 4:**
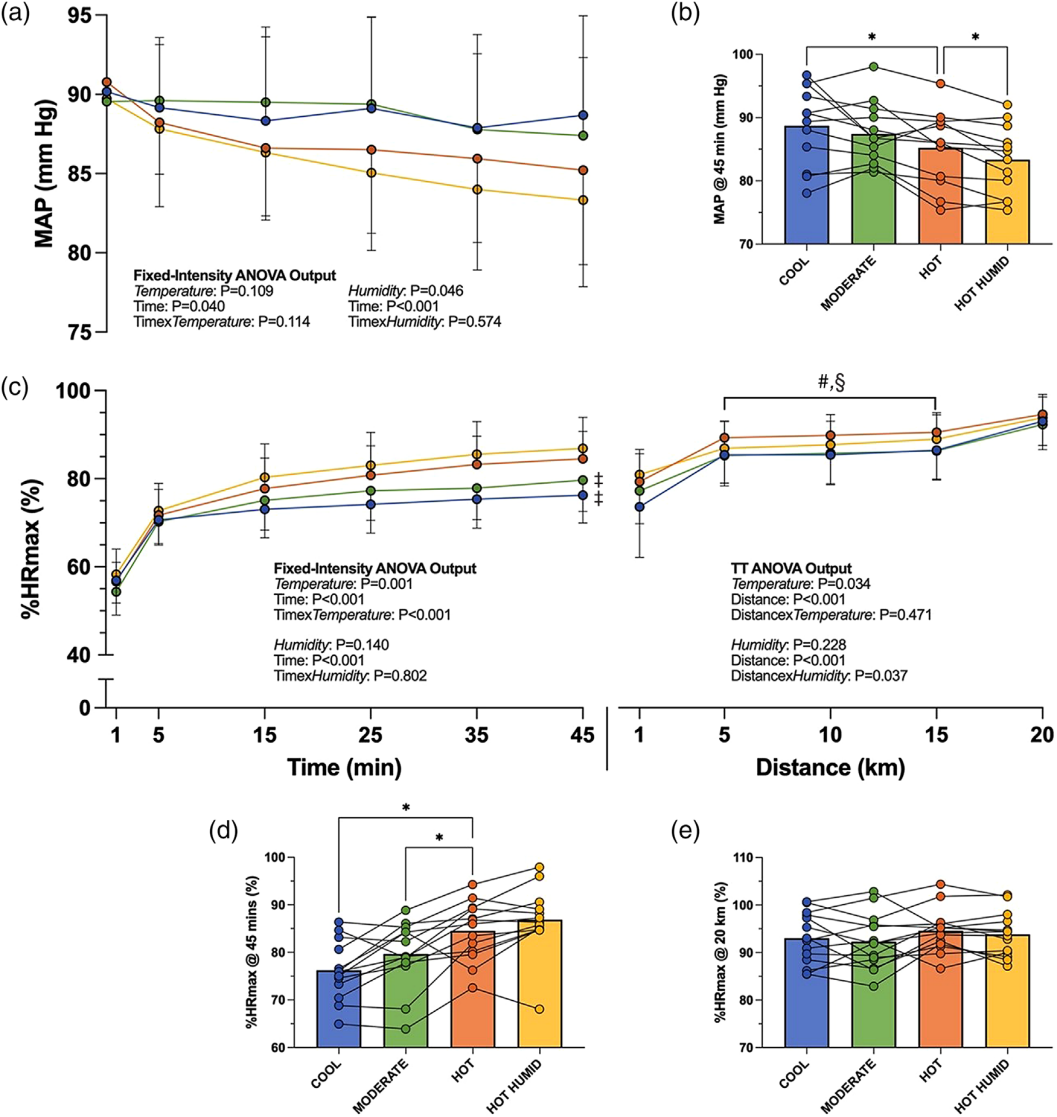
(a, b) Mean arterial blood pressure (MAP) (±SD) throughout the 45‐min fixed‐intensity period (a), with individual data shown for 45 min time point (b). (c) Mean percentage of maximum heart rate (±SD) throughout 45 min of fixed‐intensity cycling followed by a 20‐km time trial. (d, e) Individual heart rate data shown for 45 min (d) and 20 km (e) time points in respective lower panels. ^#,‡,§^Significant difference from Cool (#), Hot (‡) or Hot Humid (§) at end of 45‐min fixed‐intensity period or middle 10 km of TT (*P* < 0.05). *Significant difference between conditions (*P* < 0.05). *n* = 12 for blood pressure measures, otherwise *n* = 13 for fixed intensity, *n* = 14 for TT. %HR_max_, percentage of maximum heart rate; TT, time trial

Air temperature significantly affected heart rate (expressed as a percentage of maximum; %HR_max_) during the fixed‐intensity exercise (interaction: *P* < 0.001), such that it was higher in Hot than in Cool by the 15th minute (*P* = 0.010), and higher than in Moderate by the 35th minute (*P* = 0.026) onwards (Figure 4). Higher air temperature increased heart rate during the TT also (interaction: *P* = 0.471; temperature: *P* = 0.034), with the middle 10 km being 4% higher in Hot than in Cool (95% CI: 1, 7; *P* = 0.007) and possibly also in Moderate (95% CI: –1, 7; *P* = 0.058).

Mean %HR_max_ was not reliably affected by humidity during fixed‐intensity exercise (interaction: *P* = 0.802; humidity: *P* = 0.140) but was 2% higher during the TT (interaction: *P* = 0.037; humidity: *P* = 0.228), specifically during the middle 10 km (95% CI: 1, 3; *P* = 0.004; Figure 4).

#### 3.4.2 Blood pressure

Air temperature did not significantly affect the time‐mediated (*P* < 0.001) increase in SBP (interaction: *P* = 0.351; temperature: *P* = 0.832) or decreases in DBP (interaction: *P* = 0.098; temperature: *P* = 0.051) or MAP (interaction: *P* = 0.114; temperature: *P* = 0.109) during the fixed‐intensity period.

Humidity had no effect on SBP (interaction: *P* = 0.594; humidity: *P* = 0.804) but reduced DBP (interaction: *P* = 0.039) by 4 mmHg (95% CI: 1, 7; *P* < 0.001) within the fixed‐intensity period, and hence reduced MAP (interaction: *P* = 0.574; humidity: *P* = 0.046; Figure 4).

### 3.5 Psychophysical

#### 3.5.1 Rating of perceived exertion

**FIGURE 5:**
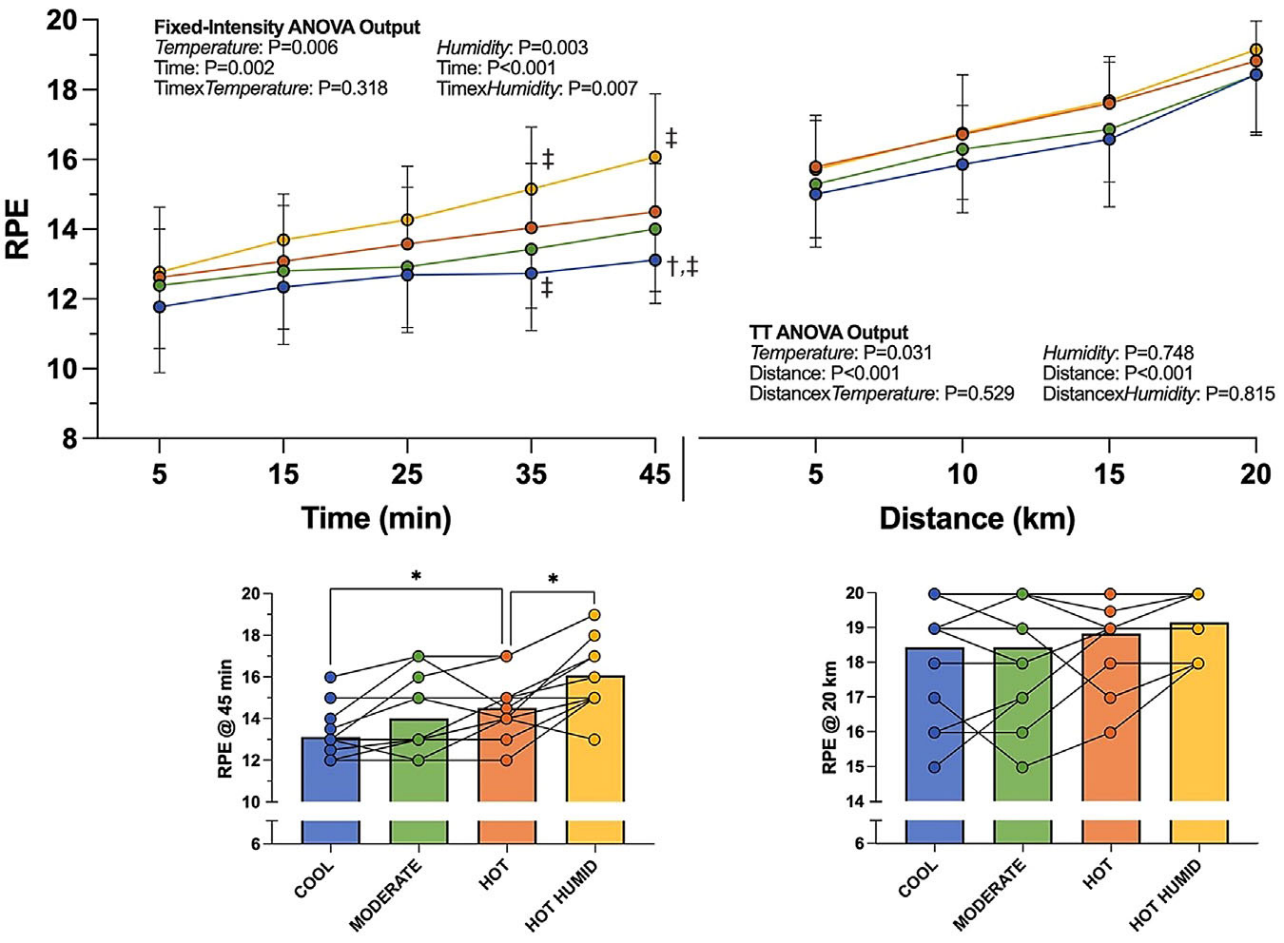
Mean rating of perceived exertion (RPE) (9 = very light, 13 = somewhat hard, 19 = extremely hard) (±SD) throughout 45 min of fixed‐intensity cycling followed by a 20‐km time trial. Individual data shown for 45 min and 20 km time points in respective lower panels. ^†,‡^Significant difference from Moderate (†) or Hot (‡) at that given time point (*P* < 0.05). *Significance difference between conditions (*P* < 0.05). *n* = 13 for fixed intensity, *n* = 14 for TT

Higher air temperature increased RPE (interaction: *P* = 0.318; temperature: *P* = 0.006), with RPE in Hot (15 ± 1) and Moderate (14 ± 2) being higher than that in Cool (13 ± 1; *P* = 0.008 and *P* = 0.040, respectively; Figure 5) by 45 min. In the TT, RPE was similar between conditions (*P* = 0.529), as would be expected.

Elevated humidity also increased RPE (interaction: *P* = 0.007; humidity: *P* = 0.003), being 2 units higher by 45 min (95% CI: 1, 2; *P* < 0.001; Figure 5). RPE was comparable between humidity conditions within the TT (interaction: *P* = 0.815; humidity: *P* = 0.748).

#### 3.5.2 Thermal perceptions and affect

**FIGURE 6:**
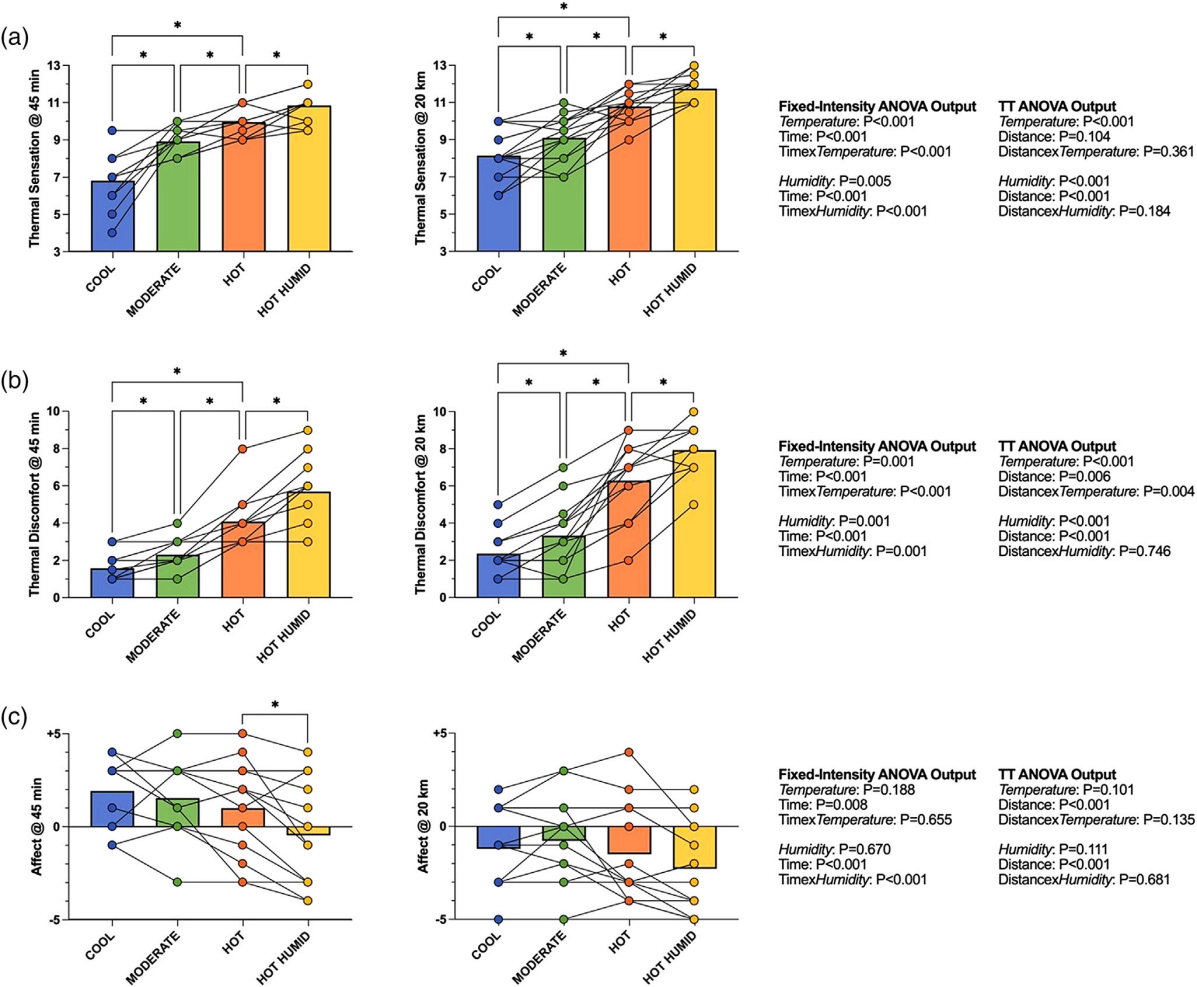
Mean and individual thermal sensation (3 = very cold, 7 = neutral, 11 = very hot) (a), thermal discomfort (1 = comfortable, 5 = uncomfortable, 9 = extremely uncomfortable) (b) and affect (–5 = very bad, 0 = neutral, +5 = very good) (c) (± SD) at 45 min and 20‐km time points. *Significant difference between conditions (*P* < 0.05). *n* = 13 for fixed intensity, *n* = 14 for TT. TT, time trial

Elevated air temperatures exacerbated both thermal sensation (interaction: *P* < 0.001) and discomfort (interaction: *P* < 0.001; Figure 6) throughout the fixed‐intensity period. These effects persisted throughout the TT, with Hot providing significantly greater thermal discomfort than Moderate (by 3; 95% CI: 2, 4; *P* < 0.001) and Cool (by 4; 95% CI: 3, 5; *P* < 0.001) at 20 km. Air temperature had no impact on affect, with feeling state worsening progressively, and to a similar extent across conditions during exercise.

Elevated humidity also elicited higher thermal sensation (interaction: *P* < 0.001) and discomfort (interaction: *P* = 0.001; Figure 6) in the fixed‐intensity period, continuing through to the TT, with the Hot Humid environment being perceived as hotter (by the 5th minute; *P* = 0.042) and more thermally uncomfortable (by the 5th minute; *P* = 0.004) than Hot. Humidity also significantly lowered participants’ feeling scale response during the fixed‐intensity period (interaction: *P* < 0.001), by –1 at 45 min (95% CI: –2, –1; *P* < 0.001). This did not persist during the TT (interaction: *P* = 0.681; humidity: *P* = 0.111).

### 3.6 Summary table

To formally test the contextual hypothesis, that is, that effects of ‘warmer environments’ would be less evident than in previous studies (wherein absolute humidity was not held constant), further analyses were run comparing Cool and Moderate to the Hot Humid environment. This enabled demonstration of how matched %RH protocols can overstate the effect of temperature on endurance exercise performance, thermal, cardiovascular and perceptual responses. These outcomes, as shown in Table 3, reveal that when absolute humidity confounds the analysis of temperature effects, mean differences in performance, thermal, cardiovascular and perceptual variables were consistently higher than the mean differences observed when absolute humidity was matched (with the exception of *T*_sk_ and sweat rate).

**TABLE 3:** Comparison of mean differences (95% confidence interval) for performance, physiological and perceptual measures between conditions matched for absolute humidity (left columns) or not (right columns)

	Matched absolute humidity, i.e., vs. Hot		Unmatched absolute humidity, i.e., vs. Hot Humid	
	Cool	Moderate	Cool	Moderate
TT performance (min)	✓ 2.2 (1.3, 3.2)	✓ 1.4 (0.3, 2.5)	✓ 3.6 (2.4, 4.8)	✓ 2.7 (1.5, 3.9)
Δ*T*_c_ (°C)	χ 0.0 (−0.3, 0.3)	χ 0.1 (−0.1, 0.3)	χ 0.3 (−0.1, 0.6)	✓ 0.4 (0.2, 0.6)
*T*_sk_ (°C)	✓ 10.0 (8.8, 10.9)	✓ 4.2 (3.7, 4.6)	✓ 10.0 (8.8, 11.0)	✓ 4.2 (3.7, 4.8)
%HR_max_ (%)	✓ 8.3 (5.1, 11.5)	✓ 4.9 (0.4, 9.4)	✓ 10.6 (6.5, 14.7)	✓ 7.2 (3.4, 11.0)
Sweat rate (L/h)	✓ 0.7 (0.4, 0.9)	✓ 0.4 (0.2, 0.5)	✓ 0.6 (0.4, 0.8)	✓ 0.3 (0.2, 0.5)
MAP (mmHg)	✓ 3.5 (−6.4, −0.6)	χ −2.2 (−5.1, 0.8)	✓ −5.4 (−8.8, −1.9)	✓ −4.1 (−6.2, −1.9)
RPE	✓ 1.4 (0.3, 2.5)	χ 0.5 (−0.6, 1.6)	✓ 3.0 (1.9, 4.0)	✓ 2.1 (1.1, 3.1)
Thermal sensation	✓ 3.2 (1.9, 4.4)	✓ 1.0 (0.4, 1.7)	✓ 4.0 (2.6, 5.5)	✓ 1.9 (1.2, 2.6)
Thermal discomfort	✓ 2.5 (1.4, 3.6)	✓ 1.8 (0.8, 2.7)	✓ 4.1 (2.7, 5.6)	✓ 3.4 (2.0, 4.8)
Affect	χ −0.9 (−2.9, 1.1)	χ −0.5 (−1.7, 0.7)	✓ −2.4 (−4.4, 0.4)	✓ −2.0 (−3.3, −0.7)

Mean differences presented as Hot – Cool and Moderate (matched absolute humidity) and Hot Humid – Cool and Moderate (unmatched absolute humidity). ✓ or χ denotes significant (*P* < 0.05) or insignificant (*P* > 0.05) difference, respectively, from Hot or Hot Humid at end of 45‐min fixed‐intensity (except for TT performance and sweat rate – different time periods). %HR_max_, percentage of maximum heart rate; MAP, mean arterial pressure; RPE, rating of perceived exertion; Δ*T*_c_, change in core temperature; *T*_sk_, skin temperature; TT, time trial.

## 4 DISCUSSION

The purpose of the present study was to delineate the independent effects of air temperature and humidity on performance, thermal, cardiovascular and perceptual responses during endurance exercise. The main findings were in line with the primary hypothesis – that elevated air temperature would increase heat strain and impair TT performance, but to a lesser extent than has been previously reported. Table 3 details the differences in temperature effects when humidity was matched and unmatched, revealing that TT performance, Δ*T*_c_, %HR_max_, MAP and perceptual responses were all higher/impaired when humidity was allowed to act as a confounding factor, as it has in almost all previous research.

TT performance was impaired in 36°C when absolute humidity was matched between conditions (Figure 2 and Table 1). Insignificant performance differences between 18 and 27°C contrast with previous research citing the mediating effects of increased *T*_sk_, namely a reduced *T*_c_ – *T*_sk_ gradient and exacerbated cardiovascular strain, as primary reasons for performance decrements in a hotter environment (Ely et al., 2010; Schlader et al., 2011; Tatterson et al., 2000). These discrepancies may reflect the greater evaporative capacity at 27°C compared to studies that matched for %RH, thus allowing for the evaporation of sweat at a higher rate, in turn mitigating reductions in the *T*_c_ – *T*_sk_ gradient, slowing heat storage and improving TT performance. However, as performance was impaired in Hot in comparison to Cool and Moderate environments, it may be that above a certain ambient temperature threshold, a superior *P*_sk,sat_ – *P*_a_ gradient is unable to compensate for the reduced *T*_c_ – *T*_sk_ gradient or increased *T*_sk_ itself, thus elevating cardiovascular strain and impairing aerobic performance. This theory is consistent with the work of Kenefick et al. (2010), who found that when *T*_sk_ was higher than 29°C, aerobic performance was degraded by ∼1.6% for every 1°C *T*_sk_ increase. TT performance was further impaired when absolute humidity was doubled at 36°C, supporting observational data linking increased humidity to slowed marathon performances in thermally stressful environments (El Helou et al., 2012; Montain et al., 2007). It is also noted, however, that when humidity was allowed to act as a confounder in the analysis of temperature effects, the mean performance decrement was 60–90% greater than when absolute humidity was matched between temperature conditions (Table 3).

Despite large *T*_sk_ differences between conditions, no significant differences in Δ*T*_c_ between humidity‐matched environments were evident during fixed‐intensity exercise (Figure 3). Similar results in Lei et al. (2020) were attributed to (1) higher evaporative capacity at the higher air temperature – due to a warmer *T*_sk_ expanding the *P*_sk,sat_ – *P*_a_ gradient, and (2) a greater contribution of dry‐heat transfer to heat balance at the lower air temperature. This may provide an explanation for results in the present study, with observed sweat rates in Hot being 2.6 and 1.5 times greater than those in Cool and Moderate, respectively. This higher evaporative heat loss in Hot may have mitigated reductions in dry heat loss (i.e., convection and radiation) during the fixed‐intensity cycle, blunting the rate of heat storage, or at least the Δ*T*_c_, so that responses across conditions were comparable. It should be noted that the duration of our fixed‐intensity period may not have been long enough to elicit significant Δ*T*_c_ at higher air temperatures, and had it been longer, larger effects may have been evident. A larger Δ*T*_c_ observed in higher humidity during the fixed‐intensity period (Figure 3 and Table 3) is consistent with previous research finding that higher humidity compromises the evaporative capacity of an environment by way of a decreased *P*_sk,sat_ – *P*_a_ gradient, such that rate of heat loss is reduced, increasing the rate of body heat storage (Maughan et al., 2012; Muhamed et al., 2016). Absences of *T*_sk_ differences observed between humidity conditions were surprising, as González‐Alonso (2012) and Sawka et al. (2012) indicate that the decline in sweating efficiency in more humid environments elevates *T*_sk_ by virtue of slower evaporation and greater skin perfusion. Furthermore, larger Δ*T*_c_ is typically accompanied by similar increases in *T*_sk_ during exercise in the heat, due to the passive and active heat transfer required for heat exchange (Cuddy et al., 2014). It is possible that the lack of *T*_sk_ differences in the present study could be attributed to markedly higher convective heat loss in the Hot Humid environment, as previous studies to have observed higher *T*_sk_ in elevated humidities have utilised minimal and no forced‐convective cooling (Maughan et al., 2012; Moyen et al., 2014), thus impairing heat loss and perhaps serving as a less ecologically valid investigation for outdoor exercise contexts.

The %HR_max_ was understandably higher in 36°C during fixed‐intensity exercise, due to a warmer *T*_sk_ driving increased skin blood flow, and a reduction in stroke volume (Cheuvront et al., 2003; Chou et al., 2018; González‐Alonso et al., 2008). The continued progression of these responses, known as cardiovascular drift (Wingo et al., 2012), may act as one of the mechanisms by which ${\dot{V}}_{O_{2}\max}$ is reduced in the heat (Lafrenz et al., 2008), providing another potential explanation for the performance decrements observed in Hot. Heart rate did not differ significantly between humidity conditions during fixed‐intensity exercise (Figure 4); however, as elevated heart rates in humid conditions are often attributed to an increased skin blood flow demand, similar %HR_max_ values may be explained by lack of difference in *T*_sk_.

Ambient temperature had no measurable effect on MAP over the course of the fixed‐intensity exercise (Figure 4). While DBP may have been expected to decrease in warmer environments (due to reduced peripheral resistance associated with higher *T*_sk_; Wyndham (1973), thus decreasing MAP, Chou et al. (2018) found that a reduced *T*_c_ – *T*_sk_ gradient (by way of increased *T*_sk_) during moderate‐intensity exercise does not affect MAP, despite a graded increase in heart rate and decrease in stroke volume. Reductions in MAP observed in higher humidity appear to have been due more to decreases in DBP than SBP. In the modes of heat stress study by Campbell et al. (2022), exercise in the heat drove the greatest decrease in MAP in the absence of warmer *T*_sk_, with differences attributed to higher *T*_c_ and the associated peripheral vasodilatation needed to offload heat. This elevated cardiovascular strain at increased humidity likely contributes to the impaired performance in the Hot Humid environment (Périard et al., 2011).

Increased air temperature elevated RPE, thermal sensation and thermal discomfort (Figures 5 and 6), with perceptual differences seemingly reflecting differences in *T*_sk_ (Gagge et al., 1969) and sweat rates (Schlader et al., 2011). Warmer *T*_sk_ is known to increase RPE at a given intensity (Maw et al., 1993), but not when self‐pacing is permitted (Tucker et al., 2004), suggesting that RPE is a regulated perceptual variable that contributes to exercise intensity selection (Schlader et al., 2011; Tucker, 2009). Elevated thermal sensation and discomfort responses may contribute to impaired performance in hotter environments, as they have been noted to alter individual motivation for continued exercise in the heat (Cotter et al., 2001), with some suggesting that they act as perceptual modulators of exercise intensity, particularly at its onset (Schlader et al., 2011). Increased humidity was also associated with an increased RPE, thermal sensation and thermal discomfort, and a decreased affect during fixed‐intensity cycling, with differences presumably attributable to an increased skin wettedness associated with the reduced evaporative capacity of the Hot Humid environment (Fukazawa & Havenith, 2009; Gagge et al., 1967). Selected exercise intensity in the heat likely reflects the combined and intricately linked effects of both physiological and perceptual responses.

### 4.1 Limitations

Further limitations should be acknowledged. Firstly, the absolute humidity in Cool was 11% (0.21 kPa) lower than its set point. While this means that the effect of air temperature was not studied entirely independently in this condition, it also generated a negative bias, that is, the effect of air temperature may be even smaller than was observed here due to the higher evaporative capacity of this environment. Secondly, drinking water, while initially served at approximately the same temperature (∼19°C) in all conditions, warmed over time when exposed to hotter environments. In the cooler trials, drinking water was a more effective heat sink, attenuating the rate of body heat storage to a greater extent than that of the 36°C environments, which in turn might improve endurance exercise performance (Mündel et al., 2006). This maintains some level of ecological validity, however, and is another negative bias. Finally, while the air velocity of 4.5 m/s was intended to improve ecological validity for outdoor aerobic exercise, especially when compared to that of prior research (e.g., Galloway & Maughan, 1997; Moyen et al., 2014), velocities utilised should ideally be matched to individual movement speed, so as to best replicate what would be the case if participants were moving through still air. Had this been possible in the present study, performance, cardiovascular strain and perceptual differences between conditions may have been reduced (Otani et al., 2018).

### 4.2 Perspectives/implications

While humidity is less important than air temperature for people at rest (e.g., administrators, coaches, trainers and spectators), findings from the present study highlight the importance of absolute humidity alongside temperature when exercising or working under severe heat stress, with athletes and outdoor workers relying disproportionately on humidity for evaporative heat loss at much higher rates. Practically, air temperature and humidity are increasing globally (Blunden & Arndt, 2019), as is exposure of athletes in major sporting events, as was seen in the Tokyo Olympics of 2020, leading to increased loss of work productivity (Romanello et al., 2021) and the number of athletes suffering mild‐to‐severe heat‐related illness (Yamasaki & Nomura, 2022). Furthermore, those at the greatest risk of excessive heat exposure, and therefore mortality, are outdoor workers whose occupation is physically demanding and necessitates greater evaporative requirements (Lucas et al., 2014). Resultantly, at‐risk individuals should consider these environmental parameters when preparing to compete or work in thermally stressful environments. Furthermore, as more and more people come to be exposed to extreme conditions, it is important for them to understand thermal behaviour and their tolerance to different environments so as to decrease their risk of heat injury.

This study used equal numbers of male and female participants to reflect the population but was not designed to test possible sex differences. Sex differences might occur, on average, due to thermodynamic effects of body composition (Corbett et al., 2020), sweat production and efficiency (Kenney, 1985), and acute sex hormone effects on central and peripheral components of thermoregulation (Lei et al., 2017), and might therefore warrant investigation. It may also be of interest for future research to investigate whether individuals with greater aerobic power are more negatively impacted by elevated humidity when exercising in uncompensable environments, comparing performance, thermal and cardiovascular responses of untrained individuals to athletes who have increased sweat production and thus have higher evaporative requirements. Similar research could be suggested for animals (e.g., dogs), which are reliant on behaviour and on evaporative heat transfer via respiratory processes to cope with heat stress.

### 4.3 Conclusions

This study is, to our knowledge, the second to investigate the effects of air temperature independent from humidity, and the first to compare the effects of temperature and humidity within the same study. It is also one of very few studies to investigate the effects of ambient heat stress on female performance within a laboratory setting. When examined independently, elevated air temperature increased heat strain and impaired aerobic exercise performance, but to a lesser extent than has been reported previously. Findings from the present study highlight the importance of absolute humidity alongside temperature when exercising or working under severe heat stress. Future research investigating the effects of temperature should aim to control for the confounding effects of humidity.

## AUTHOR CONTRIBUTIONS

Elliott J. Jenkins and James D. Cotter conceived the question. Elliott J. Jenkins, Jason K. W. Lee, Toby Mündel and James D. Cotter contributed to the design. Elliott J. Jenkins, Holly A. Campbell and James D. Cotter collected the data at the School of Physical Education, Sport and Exercise Sciences, University of Otago. Elliott J. Jenkins and James D. Cotter contributed to analysis and/or interpretation of the work and wrote the first draft of the manuscript. All authors provided critical feedback and approved the final version of this manuscript and agree to be accountable for all aspects of the work in ensuring that questions related to the accuracy or integrity of any part of the work are appropriately investigated and resolved. All persons designated as authors qualify for authorship, and all those who qualify for authorship are listed.

## CONFLICT OF INTEREST

None.

## Supporting information

Statistical Summary Document

## Data Availability

The data that support the findings of this study are available from the corresponding author upon reasonable request.
